# Mutation Rates of *TGFBR2* and *ACVR2* Coding Microsatellites in Human Cells with Defective DNA Mismatch Repair

**DOI:** 10.1371/journal.pone.0003463

**Published:** 2008-10-21

**Authors:** Heekyung Chung, Dennis J. Young, Claudia G. Lopez, Thuy-Anh T. Le, Jeffrey K. Lee, Deena Ream-Robinson, Sherry C. Huang, John M. Carethers

**Affiliations:** 1 Department of Medicine, University of California San Diego, La Jolla, California, United States of America; 2 Rebecca and John Moores Comprehensive Cancer Center, University of California San Diego, La Jolla, California, United States of America; 3 Department of Pediatrics, University of California San Diego, La Jolla, California, United States of America; 4 VA San Diego Healthcare System, San Diego, California, United States of America; University of Minnesota, United States of America

## Abstract

Microsatellite instability promotes colonic tumorigenesis through generating frameshift mutations at coding microsatellites of tumor suppressor genes, such as *TGFBR2* and *ACVR2*. As a consequence, signaling through these TGFβ family receptors is abrogated in DNA Mismatch repair (MMR)-deficient tumors. How these mutations occur in real time and mutational rates of these human coding sequences have not previously been studied. We utilized cell lines with different MMR deficiencies (*hMLH1^−/−^*, *hMSH6^−/−^*, *hMSH3^−/−^*, and MMR-proficient) to determine mutation rates. Plasmids were constructed in which exon 3 of *TGFBR2* and exon 10 of *ACVR2* were cloned +1 bp out of frame, immediately after the translation initiation codon of an enhanced GFP (EGFP) gene, allowing a −1 bp frameshift mutation to drive EGFP expression. Mutation-resistant plasmids were constructed by interrupting the coding microsatellite sequences, preventing frameshift mutation. Stable cell lines were established containing portions of *TGFBR2* and *ACVR2*, and nonfluorescent cells were sorted, cultured for 7–35 days, and harvested for flow cytometric mutation detection and DNA sequencing at specific time points. DNA sequencing revealed a −1 bp frameshift mutation (A_9_ in *TGFBR2* and A_7_ in *ACVR2*) in the fluorescent cells. Two distinct fluorescent populations, M1 (dim, representing heteroduplexes) and M2 (bright, representing full mutants) were identified, with the M2 fraction accumulating over time. *hMLH1* deficiency revealed 11 (5.91×10^−4^) and 15 (2.18×10^−4^) times higher mutation rates for the *TGFBR2* and *ACVR2* microsatellites compared to *hMSH6* deficiency, respectively. The mutation rate of the *TGFBR2* microsatellite was ∼3 times higher in both *hMLH1* and *hMSH6* deficiencies than the *ACVR2* microsatellite. The −1 bp frameshift mutation rates of *TGFBR2* and *ACVR2* microsatellite sequences are dependent upon the human MMR background.

## Introduction

The DNA MMR system consists of proteins that act in concert to recognize and coordinate repair of nucleotide base mismatches and slippage mistakes at microsatellite sequences on newly synthesized DNA [1]. In humans, MMR activity requires the proper functioning of hMutSα and hMutSβ to recognize defects, and hMutLα to coordinate repair. hMutSα (heterodimer of hMSH2 and hMSH6) recognizes single nucleotide interstrand mispairs and insertion/deletion loops (IDLs) containing 1 or 2 looped nucleotides, whereas hMutSβ (heterodimer of hMSH2 and hMSH3) recognizes IDLs containing 2 or more looped nucleotides that occur at microsatellite sequences [2]. The hMutS complexes interact with the hMutLα protein complex (heterodimer of hMLH1 and hPMS2) to coordinate excision and repair of the mispair or IDL [3]–[5]. Loss of any of the components of the MMR system inactivates or attenuates DNA repair, and is the cause of microsatellite instability (MSI) [6], [7]. Patients with germline mutations of *hMSH2*, *hMLH1*, *hMSH6*, or *hPMS2* have Lynch syndrome (formerly known as hereditary nonpolyposis colon cancer or HNPCC), the most common familial form of colorectal cancer [8]–[11]. Epigenetic inactivation of *hMLH1* through promoter hypermethylation occurs in 15–20% of sporadic colorectal cancers [12], [13]. In either instance, the resulting colorectal cancers display the phenotype of MSI observed as novel length mutations at microsatellites [7].

Microsatellites are nucleotide repeat sequences that are ubiquitous throughout the genome [14]. Rarely but significantly, microsatellites are present in the coding regions (exons) of critical growth regulatory genes and are targeted for frameshift mutation when DNA MMR is defective [15]. These frameshift mutations, which occur due to non-repair of exonic IDL, are thought to drive the pathogenesis of colorectal cancers and other MSI tumors. The type II receptor for transforming growth factor β (*TGFBR2*) has an A_10_ microsatellite within exon 3. Frameshift mutation of this polyadenine sequence truncates *TGFBR2*, making it nonfunctional for TGFβ signaling [16]. In 70–90% of colorectal cancers with MSI, *TGFBR2* is frameshift mutated at both alleles [17]. This mutation allows the tumor to escape the growth suppressive effects of TGFβ–SMAD signaling. *TGFBR2* mutation appears to be a late event in MSI adenomas and tightly correlated with progression of these adenomas to malignant carcinomas [18].

The activin type II receptor, *ACVR2*, contains polyadenine tracts at both exons 3 and 10 but only its exon 10 A_8_ tract is mutated in ∼85% of colorectal cancers with MSI [19], [20]. The biallelic frameshift mutation causes ACVR2 protein loss, and is associated with histologically poor grade tumors and significantly larger volume tumors [20], [21]. Restoration of ACVR2 in colon cancer cells causes growth suppression [22].

Colon cancer cell models highlight the relationship between defective DNA MMR and *TGFBR2* and *ACVR2* frameshift mutations. Both genes commonly have a −1 bp frameshift mutation with defective MMR. Restoration of wild type (WT) *TGFBR2* and *ACVR2* by chromosome transfer reveals growth suppression in the cells and slower growth in xenografts in nude mice [22], [23]. Interestingly, HCT116+chr3 cells, which have two mutant *hMLH1* and two mutant *TGFBR2* alleles plus one WT *hMLH1* and one WT *TGFBR2* allele, express ∼33% WT *TGFBR2* mRNA and ∼67% mutant *TGFBR2* mRNA (unpublished data). On the other hand, HCT116+chr2 cells, which have two mutant *hMLH1* and two mutant *ACVR2* alleles plus one WT *ACVR2* allele, express ∼20% WT *ACVR2* mRNA [22], suggesting a slow but steady mutation of the transferred *ACVR2* allele in *hMLH1* deficiency.

Determining mutation rates of actual human coding genes in human MMR deficiency has not been previously performed, although model systems using noncoding sequences with human cell and yeast MMR systems have been utilized [24]–[27]. To test the hypothesis that *TGFBR2* and *ACVR2* frameshift mutations are dependent on the human MMR background, we constructed EGFP plasmids in which a −1 bp frameshift mutation at coding microsatellites of *TGFBR2* exon 3 and *ACVR2* exon 10 was detected by EGFP expression in human colon cancer cells with MMR deficiency. With this new cell model, we were able to calculate a human gene mutation rate in each human MMR-deficient background, and could directly compare the mutation rate between *TGFBR2* and *ACVR2*.

## Results

### Successful cloning and stable transfection of pIREShyg2-TGFBR2-EGFP and pIREShyg2 ACVR2-EGFP plasmids

**Figure 1:**
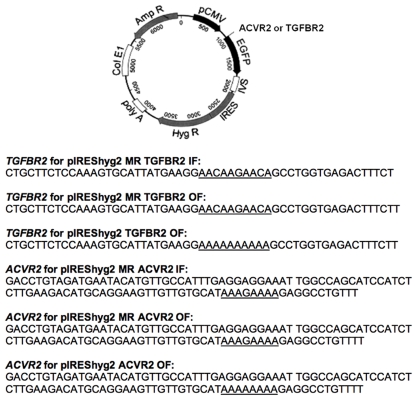
pIREShyg2-TGFBR2-EGFP and pIREShyg2-ACVR2-EGFP plasmids. Portions of exon 3 of *TGFBR2* and exon 10 of *ACVR2* sequences containing coding microsatellites were inserted immediately after the start codon of the EGFP gene, being in frame with EGFP (IF) or +1 bp out of reading frame with the EGFP (OF) in pIREShyg2-EGFP. Mutation resistant (MR) plasmids were constructed by interrupting the coding microsatellite sequences (A_10_ to A_2_CA_2_GA_2_CA in *TGFBR2* and A_8_ to A_3_GA_4_ in *ACVR2*), preventing frameshift mutation. Deletion of 1 bp in OF plasmids, experimental plasmids, restores the proper reading frame and allows EGFP expression. MR IF and MR OF plasmids were used for positive and negative control for EGFP expression, respectively.

The plasmid pIREShyg2-EGFP allows the expression of EGFP under the control of a constitutive cytomegalovirus promoter, which is active throughout the cell cycle [25]. We inserted portions of exon 3 of *TGFBR2* or exon 10 of *ACVR2* as outlined in **Fig. 1** after the translation initiation codon of the EGFP gene, either in-frame of an EGFP (IF) or +1 bp out of frame of the EGFP (OF) in pIREShyg2-EGFP. For experimental plasmids, *TGFBR2* or *ACVR2* sequences were cloned +1 bp OF in pIREShyg2-EGFP and thus a −1 bp frameshift mutation at the coding microsatellite would shift the EGFP gene into the proper reading frame to allow EGFP expression. Mutation resistant (MR) counterpart plasmids were constructed by interrupting the coding microsatellites (A_10_ to A_2_CA_2_GA_2_CA in *TGFBR2* and A_8_ to A_3_GA_4_ in *ACVR2*) and would be resistant to frameshift mutation. The MR *TGFBR2* and MR *ACVR2* plasmids were placed OF (+1 bp) and IF to be used as negative and positive controls for EGFP expression, respectively. Transient transfections of these plasmids into the *hMLH1^−/−^* background were performed to verify their EGFP expression in cells and detected by a fluorescence microscope before stable cell lines with different MMR deficiency were established. Positive controls expressed EGFP whereas negative controls did not express EGFP in the cells.

**Figure 2:**
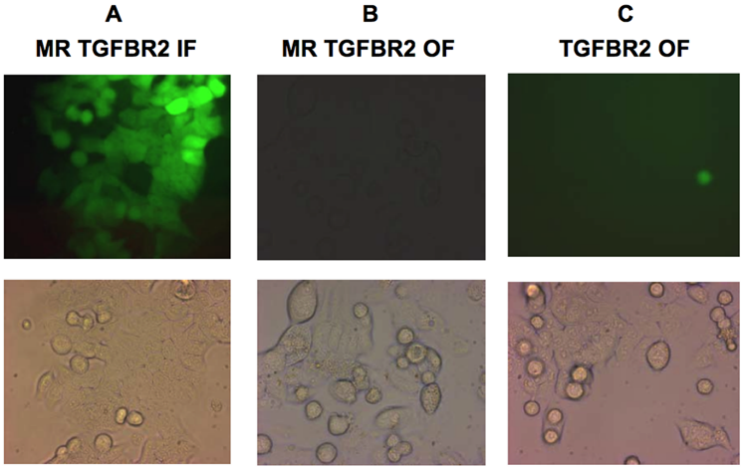
EGFP expression of stably transfected HCT116 cell lines (*hMLH1^−/−^*) containing exon 3 of *TGFBR2* sequences. (A) MR *TGFBR2* IF cells, a postive control, showed 99% EGFP expression. (B) MR *TGFBR2* OF cells, a negative control, showed no visible fluorescence. (C) *TGFBR2* OF cells showed 1.22% net fluorescence. Photomicrographs of stable HCT116 cell lines are representative of the other stable cell lines containing *TGFBR2* exon 3 or *ACVR2* exon 10 sequences. Photomicrographs in top panels were taken with a fluorescence microscope (Zeiss, Germany) and photomicrographs in bottom panels were taken with a light microscope of the identical area under 40× magnification.

Subsequently, twenty-four stable cell lines with differing MMR genetic backgrounds (**Table S1**) were established with hygromycin B selection after transfection, as outlined in **Table S2**. After selection, colonies from each cell line were initially pooled and cultured for mutation analysis. After 5 weeks, the proportion of fluorescent cells within each cell line was measured by flow cytometry. All eight cell lines containing MR *TGFBR2* IF or MR *ACVR2* IF sequence showed fluorescence between 88% and 100% (median 99.1%), indicating robust selection efficiency of the MR IF stable cell lines (**Fig. 2**). In *hMLH1^−/−^* and *hMSH6^−/−^* cells containing *TGFBR2* OF or *ACVR2* OF sequences, newly fluorescent cells were observed ranging between 0.14% and 1.22% (median 0.32%) net fluorescence over counterpart cell lines containing MR *TGFBR2* OF or MR *ACVR2* OF sequences. The *hMLH1^−/−^* cells with *TGFBR2* OF sequence showed the highest net fluorescence (1.22%) among cell lines with *TGFBR2 OF* or *ACVR2 OF* sequences (**Fig. 2**). On the other hand, *hMSH3^−/−^* and MMR proficient stable cell lines did not show any net fluorescence.

### *TGFBR2* exon 3 and *ACVR2* exon 10 coding microsatellite mutations are dependent on the MMR background

**Figure 3:**
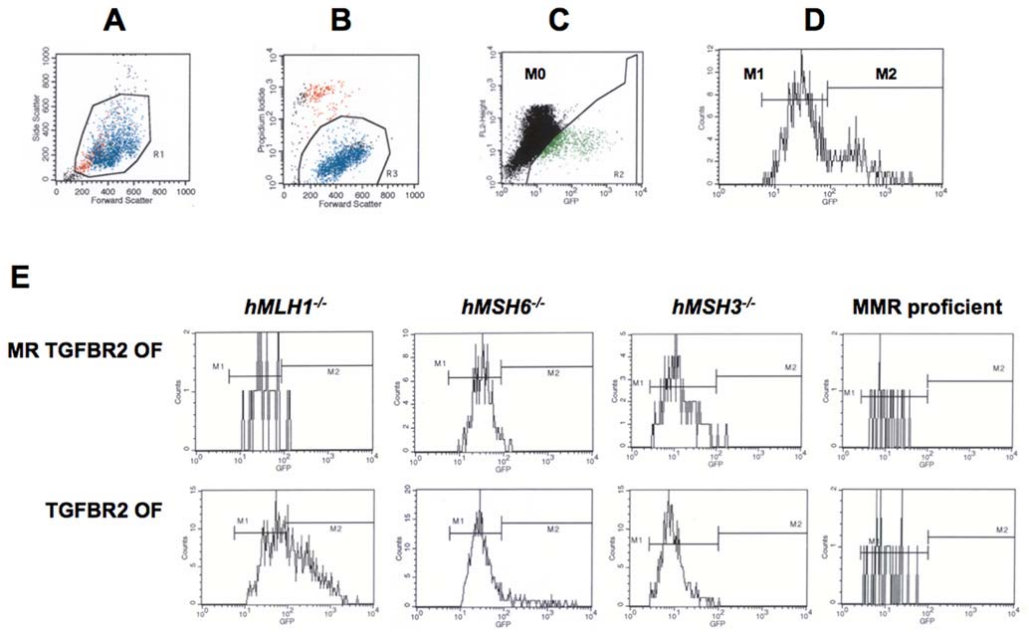
Mutation analysis by flow cytometry. Nonfluorescent cells were sorted and cells were exponentially grown for 7 to 35 days. At specific time points, cells were harvested, and 200,000 cells were analyzed for EGFP expression (identifying a −1 bp mutation) by flow cytometry. For example, with *hMLH1^−/−^ ACVR2* OF cells, region R1, R3, and R2 were set according to (A) cell size, (B) live cells, and (C) fluorescence. Gated R1 and R3 (live cells), and R2 were analyzed on an EGFP histogram (D) and two distinct EGFP populations were plotted. The population displaying no fluorescence was designated M0, the population with dim EGFP expression was designated M1, and the population with bright EGFP expression was designated M2. (E) EGFP histograms of MR *TGFBR2* OF and *TGFBR2* OF cells in different MMR deficient backgrounds at day 21 were shown as representatives of mutation analysis. Scaling of cell counts in each EGFP histogram is different for each MMR background.

To determine mutation frequencies of the *TGFBR2* and *ACVR2* coding microsatellites in cells with different MMR backgrounds (**Table S1**), nonfluorescent cells containing either MR *TGFBR2* OF, *TGFBR2* OF, MR *ACVR2* OF or *ACVR2* OF were sorted and exponentially grown for 7 to 35 days. At specific time points (day 7, 14, 21, 28, and/or 35) three cultures of each cell line were analyzed in parallel for EGFP expression by using flow cytometry to detect −1 bp frameshift mutations. Three different populations were identified according to their EGFP fluorescence intensity (**Fig. 3**). The population with no fluorescence was named M0, the population with low fluorescence M1, and the population with high fluorescence M2. EGFP histograms of MR *TGFBR2* OF and *TGFBR2* OF cells in different MMR backgrounds at day 21 are shown in **Fig. 3E**, in which *hMLH1^−/−^ TGFBR2* OF cells showed 2 distinct EGFP cell populations, M1 and M2. M2 cells from *hMLH1^−/−^ TGFBR2* OF showed brighter EGFP expression compared to M1 cells (**Fig. S1**).

**Figure 4:**
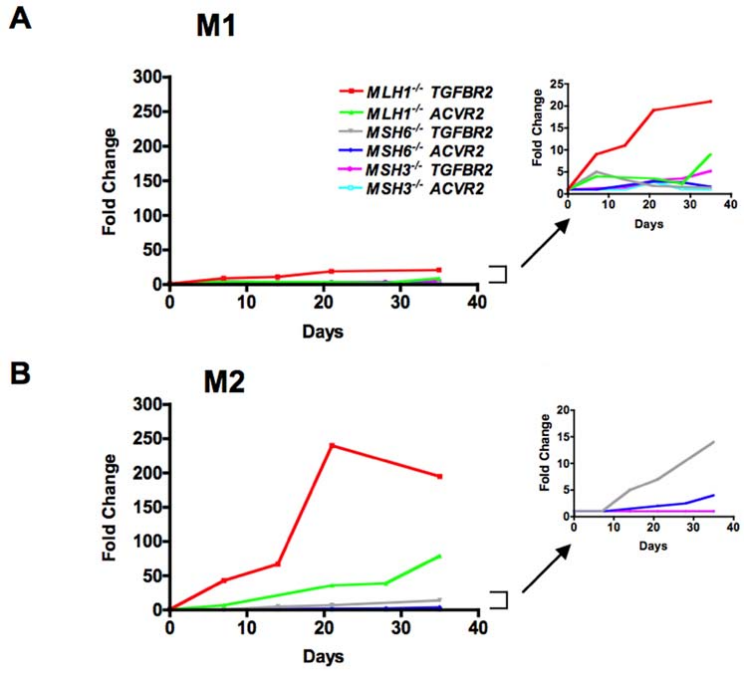
Mutation frequencies of *TGFBR2* exon 3 and *ACVR2* exon 10 are dependent on the MMR background. Nonfluorescent cells were analyzed for EGFP expression by flow cytometry at 7, 14, 21, 28, and/or 35 days after being sorted and cultured, and EGFP analysis was performed as described in Fig. 3. Mutation frequency at each time point was expressed as a fold change using the following formula: (EGFP positive cells/total live cells from *TGFBR2* OF or *ACVR2* OF cells)/(EGFP positive cells/total live cells from MR *TGFBR2* OF or MR *ACVR2* OF cells). Overall, the M1 population showed little change in mutation frequency whereas mutation frequency of the M2 population accumulated over time, indicating that M1 and M2 populations are distinct populations. In the M2 population, note that the *hMLH1* deficiency conferred higher mutation frequencies in both *ACVR2* and *TGFBR2* sequences compared to *hMSH6* and *hMSH3* deficiencies and that *TGFBR2* sequence showed a higher mutation frequency than *ACVR2* sequence in identical MMR deficiencies. Cell lines showing lower mutation frequencies (less than 25-fold change) were separately plotted in the right panel using a smaller y-axis scale. Data are means from three independent experiments at each time point.

The −1 bp mutation frequency at each time point was expressed as a fold change using the following formula: (EGFP positive cells/total live cells from *TGFBR2* OF or *ACVR2* OF cells)/(EGFP positive cells/total live cells from MR *TGFBR2* OF or MR *ACVR2* OF cells) (**Fig. 4**). The M2 population accumulated over time (most dramatically with *TGFBR2* and *ACVR2* sequences in *hMLH1^−/−^* background) whereas the M1 population showed little change (**Fig. 4**), indicating that M1 and M2 are distinct populations. The M1 and M2 populations were plotted separately for analysis of mutation frequency.

In the M1 population, mutation frequency of the *TGFBR2* sequence in *hMLH1^−/−^* cells was higher than other cell lines and increased over time (highest at day 35, 21-fold change), although the increase over time was small when compared to the increase in the M2 population (**Fig. 4A**). There was no consistent increase in the M1 population in other cell lines over time except *hMSH3^−/−^* TGFBR2 cells that showed a slow increase in mutation frequency up to day 35 (5-fold change). In the M2 population, the *TGFBR2* sequence in the *hMLH1^−/−^* background demonstrated the highest mutation frequency (highest at day 21, 240-fold change) over time compared to other different MMR deficient backgrounds as predicted in EGFP histograms (**Fig. 3E**). Mutation frequency of the *TGFBR2* sequence in the *hMSH6^−/−^* background also increased over time and showed the highest mutation frequency on day 35 (14-fold change), although this frequency is much lower than *TGFBR2* sequence in *hMLH1^−/−^* cells (**Fig. 4B**). At day 35, frameshift mutation of *ACVR2* sequence in the *hMLH1^−/−^* background was 79-fold higher than the negative control whereas *ACVR2* sequence in the *hMSH6^−/−^* background showed a 4-fold change in mutation frequency. In all *hMSH3^−/−^* stable cell lines, M2 cells were rare events (average 0.009%) at all time points and there was no significant difference in numbers of the M2 population between MR *TGFBR2* or MR *ACVR2* OF and *TGFBR2* or *ACVR2* OF cells, respectively, as shown in **Fig. 3E**. Thus, fold changes in the M2 population were 1 at all time points for both *TGFBR2* and *ACVR2* sequences in *hMSH3^−/−^* cell lines (**Fig. 4B**).

### The M1 population represents pass-through heteroduplexes, while the M2 population represents fully mutant sequences

**Figure 5:**
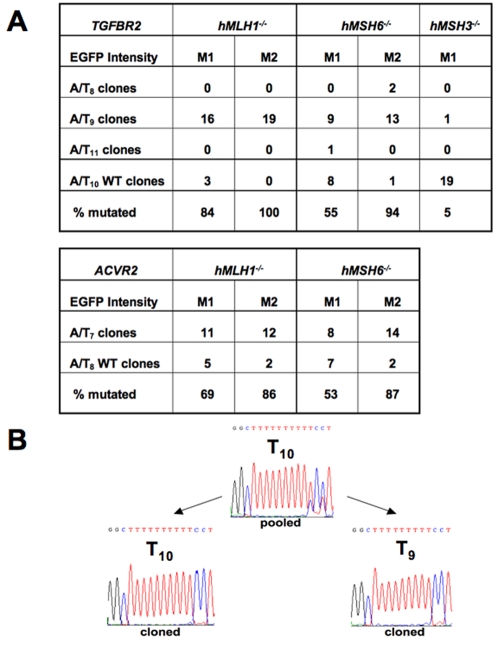
Frameshift mutation at coding microsatellites of *TGFBR2* exon 3 and *ACVR2* exon 10 in different human MMR deficient backgrounds. Cells from the M1 and/or M2 populations of *hMLH1^−/−^ TGFBR2* OF or *ACVR2* OF cells, *hMSH6^−/−^ TGFBR2* OF or *ACVR2* OF cells, and *hMSH3^−/−^ TGFBR2* OF cells were sorted and cultured. DNA from each cell line was amplified by PCR, sub-cloned and all single cell clones were individually sequenced to assess for frameshift mutation of the coding microsatellites of *TGFBR2* exon 3 and *ACVR2* exon 10. Sequence analysis of DNA clones from *hMLH1* and *hMSH6* deficiencies revealed mostly 1 bp deletion at microsatellites (A_9_ for *TGFBR2* or A_7_ for *ACVR2*), shifting the EGFP gene into the reading frame and leading to its expression (A). Note that M2 clones from *hMLH1^−/−^* TGFBR2 OF cells revealed 100% A_9_/T_9_ microsatellite sequences, termed a “full mutant” whereas M1 clones revealed a mixture of A_10_/T_10_ and A_9_/T_9_ microsatellite sequences, which suggests the presence of an A_10_/T_9_ heteroduplex, termed an “intermediate mutant” (B).

To confirm that fluorescence from the M1 and M2 populations was driven by −1 bp frameshift mutation at the coding microsatellites of *TGFBR2* OF and *ACVR2 OF*, at day 21 after being plated as nonfluorescent cells, cells from the M1 and/or M2 populations of *hMLH1^−/−^ TGFBR2* OF or *ACVR2* OF cells, *hMSH6^−/−^ TGFBR2* OF or *ACVR2* OF cells, and *hMSH3^−/−^ TGFBR2* OF or *ACVR2* OF cells were sorted and expanded for sequencing analysis. DNA from each cell line was amplified by PCR, sub-cloned into a TA cloning vector and single cell clones were individually sequenced to assess for frameshift mutation at the coding microsatellites of *TGFBR2* exon 3 and *ACVR2* exon 10. As expected, nearly all DNA clones (86–100%) from the M2 population of all cell lines with *hMLH1^−/−^* and *hMSH6^−/−^* revealed −1 bp frameshift mutation (A_9_ at *TGFBR2* and A_7_ at *ACVR2*), indicating fully mutant sequences inducing EGFP expression (**Fig. 5A**). Rare A_8_ sequences were observed in M2 clones from *hMSH6^−/−^ TGFBR2* OF cells. In particular, all clones from the M2 population of *hMLH1^−/−^ TGFBR2* OF cells revealed frame shift mutations (A_9_) with no wild type A_10_ sequence (**Fig. 5A**). This observation correlates with the highest mutation frequency of *hMLH1^−/−^ TGFBR2* cells over time (**Fig. 4B**). In comparison, clones from the M1 population of *hMLH1^−/−^ TGFBR2* OF and *hMLH1^−/−^ ACVR2* OF revealed 84 and 69% of mutant (A_9_ and A_7_) microsatellites, respectively, and clones from the M1 population of *hMSH6^−/−^ TGFBR2* OF and *hMSH6^−/−^ ACVR2* OF cells expressed 50 and 53% of mutant (A_9_ and A_7_) microsatellites, respectively (**Fig. 5A**). A rare A_11_ sequence was also observed in M1 clones from *hMSH6^−/−^ TGFBR2* OF cells. In the M1 population, clones of *hMLH1^−/−^ TGFBR2* OF cells showed a −1 bp frameshift mutation in 84%, corresponding to the highest increase in mutation frequency in the M1 population of all cell lines over time (**Fig. 4A**). Only five percent (1/20) of M1 clones from *hMSH3^−/−^ TGFBR2* OF cells revealed a mutated microsatellite sequence (A_9_) (**Fig. 5A**). The M1 population from *hMSH3^−/−^ ACVR2* OF cells did not show any frameshift mutation (data not shown) and thus sub-cloning was not done for sequencing analysis. As expected, all MR stable cell lines did not show frameshift mutations at microsatellites with MMR-deficiency. In addition, all MMR proficient HT29 stable cell lines did not show any frameshift mutations at microsatellites of *TGFBR2* and *ACVR2*.

To determine the nature of the mutations observed in the M1 population, we analyzed pooled cells as well as single cell clones. In pooled samples, unlike fully mutant sequences observed in the M2 populations, M1 population sequences often revealed two overlapping sequences, suggestive of heteroduplexes (A_9_/T_10_ in *TGFBR2* and A_7_/T_8_ in *ACVR2*) (**Fig. 5B**). Single cell clones revealed the presence of both WT and −1 bp frameshift mutants (**Fig. 5B**), consistent with heteroduplexes that weakly drive EGFP expression.

Overall, our data indicate that *hMLH1* deficiency has the highest susceptibility for frameshift mutation at the coding microsatellites of *TGFBR2* exon 3 and *ACVR2* exon 10 of the three different MMR deficiencies (*hMLH1* ^−/−^, *hMSH6* ^−/−^ and *hMSH3* ^−/−^). In addition, the coding microsatellite of *TGFBR2* exon 3 has a higher susceptibility to a 1 bp frameshift mutation than that of *ACVR2* exon 10 in *hMLH1*, *hMSH6*, (and *hMSH3*) deficiencies.

### The frameshift mutation rates at the coding microsatellites of *TGFBR2* exon 3 and *ACVR2* exon 10 are dependent on the MMR background

The M2 population (full mutants) was used to calculate the mutation rates at microsatellites of *TGFBR2* exon 3 and *ACVR2* exon 10 by the “method of the mean” [28] (**Table 1**). A single mutation rate was calculated by taking a weighted average of the mutation rates at the different time points, the weights of which were chosen to minimize the variance of the estimate as previously described [25]. As predicted, the mutation rate at the microsatellite of *TGFBR2* in the *hMLH1^−/−^* background was highest: 5.91×10^−4^±1.26×10^−4^. Mutation at the A_10_ microsatellite of *TGFBR2* is ∼3 times more frequent than mutation at the A_8_ microsatellite of *ACVR2* in *hMLH1* deficiency (P<0.01). In addition, mutation at the microsatellite of *TGFBR2* is ∼4 times higher than mutation at the microsatellite of *ACVR2* in *hMSH6* deficiency. Furthermore, mutations at the microsatellites of *TGFBR2* and *ACVR2* are ∼11–15 times higher in *hMLH1* deficiency than in *hMSH6* deficiency (P<0.01). Mutation rates from *hMSH3^−/−^* and MMR-proficient cell lines were not calculated due to a lack of net fluorescent M2 populations. These data confirm that *hMLH1* deficiency allows a higher susceptibility for mutation at the coding microsatellites of *TGFBR2* exon 3 and *ACVR2* exon 10 than *hMSH6* and *hMSH3* deficiencies, and that *TGFBR2* exon 3 has a higher susceptibility to mutation at its coding microsatellite over *ACVR2* exon 10 in both *hMLH1* and *hMSH6* deficiencies.

**Table 1:** Calculated mutation rates at coding microsatellites of *TGFBR2* exon3 and *ACVR2* exon 10 in cells with MMR deficiency.

MMR Background	Gene	Microsatellite	Rate for mutation
*hMLH1^−/−^*	*TGFBR2*	A_10_→A_9_	5.91×10^−4^±1.26×10^−4^ *
*hMLH1^−/−^*	*ACVR2*	A_8_→A_7_	2.18×10^−4^±0.22×10^−4^^†^
*hMSH6^−/−^*	*TGFBR2*	A_10_→A_9_	0.54×10^−4^±0.18×10^−4^
*hMSH6^−/−^*	*ACVR2*	A_8_→A_7_	0.14×10^−4^±0.04×10^−4^

Data from the M2 cell population from each time point between day 14 and day 35 were used for mutation rate analysis. Single mutation rates were calculated by combining and averaging time-specific mutation rates. Rates are expressed as mutations at microsatellite sequence per cell per generation. Data shown are mean±SEM. *P<0.01 comparing *hMLH1^−/−^ TGFBR2* with each of *hMLH1^−/−^ ACVR2*, *hMSH6^−/−^ TGFBR2*, and *hMSH6^−/−^ ACVR2*. **^†^** P<0.01 comparing *hMLH1^−/−^ ACVR2* with each of *hMSH6^−/−^ TGFBR2*, and *hMSH6^−/−^ ACVR2*.

## Discussion

In this study, we developed an experimental model in which the actual human coding sequences of *TGFBR2* exon 3 and *ACVR2* exon 10 were evaluated in real time for −1 bp frameshift mutations in human cells with differing MMR genetic backgrounds (*hMLH1^−/−^*, *hMSH6^−/−^*, *hMSH3^−/−^*, and MMR-proficient). Our aim was to test the hypothesis that the frequency and rate of targeted genes for frameshift mutation in human MSI tumors are dependent on the MMR genetic background. −1 bp frameshift mutations in exon 3 of *TGFBR2* and exon 10 of *ACVR2* are common in MSI tumors, and are thought to help drive the pathogenesis of colorectal cancers manifesting MSI. Although the general frequencies of *TGFBR2* and *ACVR2* mutations are culled from general colorectal cancer cohorts, there is no experimental data on mutation rates of these targeted genes and how MMR deficiency can influence those rates.

In this study, we made several unique observations: (1) the −1 bp frameshift mutations at coding microsatellites within human *TGFBR2* exon 3 and *ACVR2* exon 10 sequences were observed in real time in different human MMR deficient backgrounds; (2) both coding microsatellites of *TGFBR2* and *ACVR2* mutate through heteroduplex formation (M1) before full mutation (M2); (3) MMR-deficient backgrounds determine the mutation frequency and rate of the coding microsatellites of *TGFBR2* and *ACVR2*, for which *hMLH1*>*hMSH6*>*hMSH3* deficiency; (4) *hMSH3^−/−^* background does not generate any significant frameshift mutation in the tested sequences; and (5) the coding A_10_ microsatellite of *TGFBR2* mutates at a higher rate than the A_8_ coding microsatellite of *ACVR2* in *hMLH1^−/−^* and *hMSH6^−/−^* backgrounds.

Our experimental model revealed two distinct fluorescent populations of mutant cells, M1 expressing dim EGFP and M2 expressing bright EGFP (**Fig. 3**). The M2 population accumulated over time whereas the M1 population showed little change (**Fig. 4**). These observations were similar to a study that observed frameshift mutation at a noncoding (CA)_13_ microsatellite in an *hMLH1^−/−^* background [25].

We confirmed that EGFP expression from M1 and M2 populations was driven as a result of a −1 bp frameshift mutation of *TGFBR2* OF and *ACVR2* OF cells by TA subcloning and DNA sequencing analysis (**Fig. 5**). In particular, the M2 clones from *hMLH1^−/−^ TGFBR2* OF cells revealed that all clones underwent −1 bp frameshift mutation, indicating that the M2 clones are fully mutant cells containing a frameshifted A_9_/T_9_ microsatellite. The M2 clones from *hMSH6^−/−^ TGFBR2* OF, *hMLH1^−/−^ ACVR2* OF, and *hMSH6^−/−^ ACVR2* OF cells showed ∼10–15% WT microsatellite sequences that are derived from the M1 cell population. Clones from M1 populations in *hMLH1^−/−^* and *hMSH6^−/−^* backgrounds revealed the coexistence of mutated (65±14%, A_9_ for *TGFBR2* and A_7_ for *ACVR2*) and WT (35±14%, A_10_ and A_8_) microsatellite sequences, indicating the existence of intermediate mutant cells containing A_8_/T_7_ or A_10_/T_9_ heteroduplexes within the M1 population transferring into full mutant cells as previously observed [25]. Relative to the M2 population, the M1 population increased initially but reached a steady state as a constant supply of actively mutating cells transitioned into the M2 population. Although ∼5% of cells in *hMSH3^−/−^* TGFBR2 revealed mutated microsatellite sequence (A_9_) in the M1 population, none transitioned into the M2 population. This is likely due to repair by hMutSα at the IDL, for which hMutSβ is not needed for repair.

As we hypothesized, the mutational frequencies and rates of *TGFBR2* exon 3 and *ACVR2* exon 10 microsatellites are dependent on the MMR deficient background with *hMLH1^−/−^*>*hMSH6^−/−^*>*hMSH3^−/−^*. As *TGFBR2* and *ACVR2* mutations may drive the pathogenesis of colorectal cancers, our human data is consistent with the virulence of tumor formation in Lynch syndrome. Patients with germline mutation in *hMLH1* may present with cancer at younger ages compared to those with a *hMSH6* germline mutation [29]. Data on mutation rates for *TGFBR2* exon 3 and *ACVR2* exon 10 (**Table 1**) showed similar results. *hMLH1^−/−^* TGFBR2 showed the highest mutation rate at its coding microsatellite sequence (5.91×10^−4^). This mutation rate is similar to that calculated for noncoding (CA)_13_ microsatellites in *hMLH1^−/−^* cells [25], suggesting that this coding A_10_ and the noncoding (CA)_13_ microsatellites are equally vulnerable to *hMLH1* deficiency. The mutation rate for *TGFBR2* was 3 fold higher than that for *ACVR2* in both *hMLH1* (5.91×10^−4^) and *hMSH6* (0.54×10^−4^) deficiencies. The rapid rate for *TGFBR2* mutation with MMR deficiency might be due partly to *TGFBR2*'s longer polyadenine tract compared to *ACVR2*, as longer microsatellite tracts mutate more frequently in MMR deficiency [7]. In the case of *ACVR2* exon 10, even though the mutation rate is slower than *TGFBR2* exon 3, ultimately fully mutant clones accumulate. The rapid rate for *TGFBR2* exon 3 mutation is probably most reflective in the M1 population, as there is a rapid heteroduplex formation particularly in *hMLH1* deficiency, followed by full mutation. In *ACVR2* exon 10, heteroduplex formation is relatively slower. With both *TGFBR2* and *ACVR2* constructs, heteroduplex formation and subsequent full mutation are slower in the *hMSH6^−/−^* background compared to *hMLH1^−/−^* background. It has been shown that *MSH6* and *MSH3* are redundant in regard to frameshift mutagenesis in a yeast model [30], which supports our finding that *hMSH6* and *hMSH3* defects have much lower frameshift mutation rates than the *hMLH1* defect that completely eliminates MMR. Lower frameshift mutation rate in *hMSH6* deficiency would logically predict a lower penetrance in Lynch syndrome for which no germline *hMSH3* mutation has been reported.

In summary, we established and utilized a cell model in which actual human coding microsatellite sequences of *TGFBR2* exon 3 and *ACVR2* exon 10 were evaluated in real time for frameshift mutation in different human MMR backgrounds. *hMLH1* deficiency confers a significantly higher mutation rate at the coding microsatellites of *TGFBR2* and *ACVR2* compared to *hMSH6* and *hMSH3* deficiencies. In addition, *TGFBR2* mutates at a higher rate than *ACVR2* in both *hMLH1* and *hMSH6* deficiencies. These bona-fide human genes targeted for mutation in MMR deficiency mutate at differing rates, and lose expression of their encoded proteins in colonic neoplastic cells. Understanding these targeted genes in MMR deficiency has implications in understanding the pathogenesis of MSI colorectal tumors.

## Materials and Methods

### Cloning of pIREShyg2-TGFBR2-EGFP and pIREShyg2-ACVR2-EGFP plasmids

Plasmid pIREShyg2-EGFP was a kind gift from C. Richard Boland, MD (Baylor Univ. Med Center, Dallas, TX). Details of cloning of pIREShyg2-EGFP were previously described [24]. Portions of exon 3 of *TGFBR2* and exon 10 of *AVCR2* (shown in Fig. 1) were amplified by PCR from the MMR proficient human colon carcinoma cell line FET (kind gift of Michael Brattain, Ph.D. Roswell Park Cancer Inst; Buffalo, NY). New *Pme*I and *Asc*I sites were created in the 5′ and 3′ ends of those *TGFBR2* and *ACVR2* sequences by PCR, respectively (primers: 5′-GCGTCGTTTAAACCTGCTTCTCCAAAGTGCATTATG-3′ and 5′-AAGGCGCGCCAAGAAAGTCTCACCAGGCTT-3′ for *TGFBR2* and 5′- AGCTTTGTTTAAACGACCTGTAGATGAATACATGT-3′ and 5′-AAGGCGCGCCAAACAGGCCT CTTTTTTTTATG-3′ for *ACVR2*). The PCR products and pIREShyg2-EGFP were digested with *Pme*I and *Asc*I (New England Biolabs, Ipswich, MA) and the digested PCR products were cloned into *Pme*I–*Asc*I sites of pIREShyg2-EGFP to generate pIREShyg2-TGFBR2-EGFP and pIREShyg2-ACVR2-EGFP plasmids (Fig. 1). Experimental plasmids were constructed in which the *TGFBR2* and *ACVR2* sequences were cloned +1 bp OF in pIREShyg2-EGFP immediately after the translation initiation codon of the EGFP gene, and thus frameshift mutation of −1 bp would allow expression of EGFP (Fig. 1). As negative control plasmids for EGFP expression, mutation resistant (MR) counterpart plasmids (+1 bp OF plasmids) were constructed by changing 1 or 3 nucleotide sequences (A_10_ to A_2_CA_2_GA_2_CA in *TGFBR2* and A_8_ to A_3_GA_4_ in *ACVR2*) within microsatellites using a Quickchange II site-directed mutagenesis kit (Stratagene, La Jolla, CA), preventing any frameshift mutation (Fig. 1). MR IF plasmids containing portions of *TGFBR2* or *ACVR2* were constructed as positive controls for EGFP expression (Fig. 1). The ligation products were transformed into DH5α cells. Positive colonies were screened, and the correct sequences of *TGFBR2* and *ACVR2* were confirmed by sequencing in an ABI 3700 analyzer.

### Cell lines, transfection, and selection

The human colon cancer cell lines, HT29 (MMR proficient), HCT116 (*hMLH1^−/−^* and *hMSH3^−/−^*), and DLD-1 (*hMSH6^−/−^*) were obtained from American Type Culture Collection (Rockville, MD) and maintained in either Dulbeeco's modified Eagle's medium (DMEM, Invtrogen Corp, Carlsbad, CA, for HT29 cells) or Iscove's modified Dulbeeco's medium (IMDM, Invitrogen Corp, for HCT116 and DLD-1 cells) with 10% fetal bovine serum (FBS) and penicillin (100 U/ml)/streptomycin (100 µg/ml) (P/S, Invitrogen Corp) as supplements. The HCT116 cell line containing transferred chromosome 3 (HCT116+chr3, *hMLH1* restored but *hMSH3^−/−^*) was developed as previously described [6] and maintained in IMDM containing 10% FBS, P/S, and 400 µg/ml of G418 sulfate (CellGro, Manassas, VA). Cells were transfected with various pIREShyg2-TGFBR2-EGFP and pIREShyg2-ACVR2-EGFP plasmids by using Nucleofector kit V and L (Amaxa, Cologne, Germany), following the manufacturer's instructions. Selection with hygromycin B (Invitrogen Corp) was started at 24 hr after nucleofection to generate stable cell lines. After selection, colonies from each cell line were initially pooled and cultured for mutation analysis. All stable cell lines were confirmed by sequencing.

### Analysis of mutant cells by flow cytometry

Five thousand nonfluorescent cells expressing MR *TGFBR2* OF, *TGFBR2* OF, MR *ACVR2* OF, or *ACVR2* OF were sorted into 24-well plates on a FACS ARIA by using Diva software (Becton Dickinson Immunocytometry Systems (BDIS), San Jose, CA). During a 7 to 35 day analysis period, cultures were expanded as required to keep cells in exponential growth. Cells were trypsinized, washed in PBS, and resuspended in a total volume of 200 µl of PBS/0.5 µg/ml of propidium iodide (PI) and 3% BSA. Cell suspensions were analyzed on a FACSCalibur with CELLQUEST acquisition and analysis software (BDIS, CA). At specified time points, three cultures were analyzed in parallel. To identify EGFP-positive cells, region 1 (R1) was set in the forward/side scatter and region 3 (R3) was set in the forward/PI scatter, and then R1 and R3 were gated by live cells. Region 2 (R2) was set in the fluorescence 1 (FL1, green)/fluorescence 2 (FL2, red) scatter. Cells from the gated R1, R3, and R2 were plotted further on a fluorescence intensity histogram, and three populations were separated. The population displaying no fluorescence was named M0, the population with low fluorescence intensity, M1, and the one with high fluorescence intensity, M2. The counts of M1 and M2 cells were expressed as percentages of R3 (total live cell number).

### PCR and DNA sequencing

Total cellular DNA from stable cell lines and M1 and M2 cell populations were PCR-amplified by specific primers (5′-GCGTCGTTTAAACCTGCTTCTCCAAAGTGCATTATG-3′ and 5′-TGCCGTCGTCCTTGAAGAAGA-3′ for exon 3 of *TGFBR2* and 5′- GATCCGCCACCATGTTTAAACGAC-3′ and 5′-GCTGTTGTAGTTGTACTCCAGCTTG-3′ for exon 10 of *ACVR2*) in a reaction containing the primers, buffer, DNA template, deoxynucleotides, and *Pfu* Ultra high fidelity DNA polymerase (Stratagene). The PCR products were used for DNA sequencing to identify stable cell lines and frameshift mutations at coding microsatellites. In addition, we subcloned PCR-amplified *TGFBR2* and *ACVR2* DNA fragments from M1 and M2 cell populations utilizing a TA cloning vector (Invitrogen Corp) as per the manufacturer's protocol. DNA clones were then individually sequenced to determine the prevalence of mutated and WT *TGFBR2* and *ACVR2* sequences.

### Determination of −1 bp frameshift mutation rates of *TGFBR2* exon 3 and *ACVR2* exon 10 in human cells

The mutation rate was defined as the probability of a cell undergoing a mutation in its lifetime and expressed per cell per generation. We used a “method of the mean” developed by Luria and Delbruck [28] to estimate mutation rate. The “method of the mean” is moment-based, whereby the mutation rate is estimated as a function of the sample mean of the number of mutants. The formula used in the computation is r̂ = μ*N* ln(μ*NC*), where r̂ is the mean number of mutants in a culture, *C* is the number of parallel cultures, μ is the mutation rate, and *N* is the number of cells at risk of undergoing a mutation, which Luria–Delbruck assumed to be equal to the final number of cells in a culture. Three parallel cultures were used, and r̂ was estimated as the mean of the number of mutants across the three cultures. The total number of cells *N* was based on averaging across cultures. The formula listed above was used to calculate mutation rates of the M2 cell population (full mutants) using data from flow cytometry analysis at each time point between day 14 and day 35. Single mutation rates were then calculated by combining and averaging time-specific mutation rates to minimize the variance of the estimate as previously described [25]. Data were expressed as mean±the standard errors of mean (SEM).

### Statistical analysis

Mutation rates of cell lines were compared by T-test or one-way ANOVA.

## Supporting Information

Table S1 MMR genetic background of cell lines.
(0.02 MB DOC)

Table S2 Stable cell lines expressing exon 3 of TGFBR2 and exon 10 of ACVR2.
(0.02 MB DOC)

Figure S1 M2 cells from hMLH1−/− TGFBR2 OF showed brighter EGFP expression than counterpart M1 cells.
(0.42 MB DOC)
