# Supplementary material for: Mutation Rates of TGFBR2 and ACVR2 Coding Microsatellites in Human Cells with Defective DNA Mismatch Repair
Source: PLoS One. 2008 Oct 21;3(10):e3463. doi: 10.1371/journal.pone.0003463 (PMC2565065; doi:10.1371/journal.pone.0003463)
Supplement: Table S1 — MMR genetic background of cell lines. (0.02 MB DOC) [file pone.0003463.s001.doc]

**Table S1.** **MMR genetic background of cell lines.**

| Cells MMR status MMR defect |
| --- |
| HCT116 defective *hMLH1^-/-^*, (*hMSH3^-/-^*)    HCT116 + chr3 defective  *hMSH3^-/-^* (corrected for *hMLH1*^-/-^)  DLD-1 defective  *hMSH6^-/-^*  HT29 proficient none |
