# Supplementary material for: Mutation Rates of TGFBR2 and ACVR2 Coding Microsatellites in Human Cells with Defective DNA Mismatch Repair
Source: PLoS One. 2008 Oct 21;3(10):e3463. doi: 10.1371/journal.pone.0003463 (PMC2565065; doi:10.1371/journal.pone.0003463)
Supplement: Table S2 — Stable cell lines expressing exon 3 of TGFBR2 and exon 10 of ACVR2. (0.02 MB DOC) [file pone.0003463.s002.doc]

**Table S2. Stable cell lines expressing exon 3 of *TGFBR2* and exon 10 of *ACVR2*.** pIREShyg2-mutation resistant (MR) *TGFBR2* in frame of an EGFP (IF) or MR *ACVR2* IF, MR *TGFBR2* out of frame of an EGFP (OF) or MR *ACVR2* OF, or *TGFBR2* OF or *ACVR2* OF plasmids were transfected into HCT116, DLD-1, HCT116+chr3, and HT29 cells and the cells were selected by Hygromycin B to generate stable cell lines expressing each construct.

| Cell lines expressing exon 3 of *TGFBR2* | Cell lines expressing exon 10 of *ACVR2* |
| --- | --- |
| HCT116 MR *TGFBR2* IF  HCT116 MR *TGFBR2* OF  HCT116 *TGFBR2* OF  DLD-1 MR *TGFBR2* IF  DLD-1 MR *TGFBR2* OF  DLD-1 *TGFBR2* OF  HCT116+chr3 MR *TGFBR2* IF  HCT116+chr3 MR *TGFBR2* OF  HCT116+chr3 *TGFRB2* OF  HT29 MR *TGFBR2* IF  HT29 MR *TGFBR2* OF  HT29 *TGFBR2* OF | HCT116 MR *ACVR2* IF  HCT116 MR *ACVR2* OF  HCT116 *ACVR2* OF    DLD-1 MR *ACVR2* IF  DLD-1 MR *ACVR2* OF  DLD-1 *ACVR2* OF    HCT116+chr3 MR *ACVR2* IF  HCT116+chr3 MR *ACVR2* OF  HCT116+chr3 *ACVR2* OF  HT29 MR *ACVR2* IF  HT29 MR *ACVR2* OF  HT29 *ACVR2* OF |
