# Supplementary material for: Mutation Rates of TGFBR2 and ACVR2 Coding Microsatellites in Human Cells with Defective DNA Mismatch Repair
Source: PLoS One. 2008 Oct 21;3(10):e3463. doi: 10.1371/journal.pone.0003463 (PMC2565065; doi:10.1371/journal.pone.0003463)
Supplement: Figure S1 — M2 cells from hMLH1−/− TGFBR2 OF showed brighter EGFP expression than counterpart M1 cells. (0.42 MB DOC) [file pone.0003463.s003.doc]

**
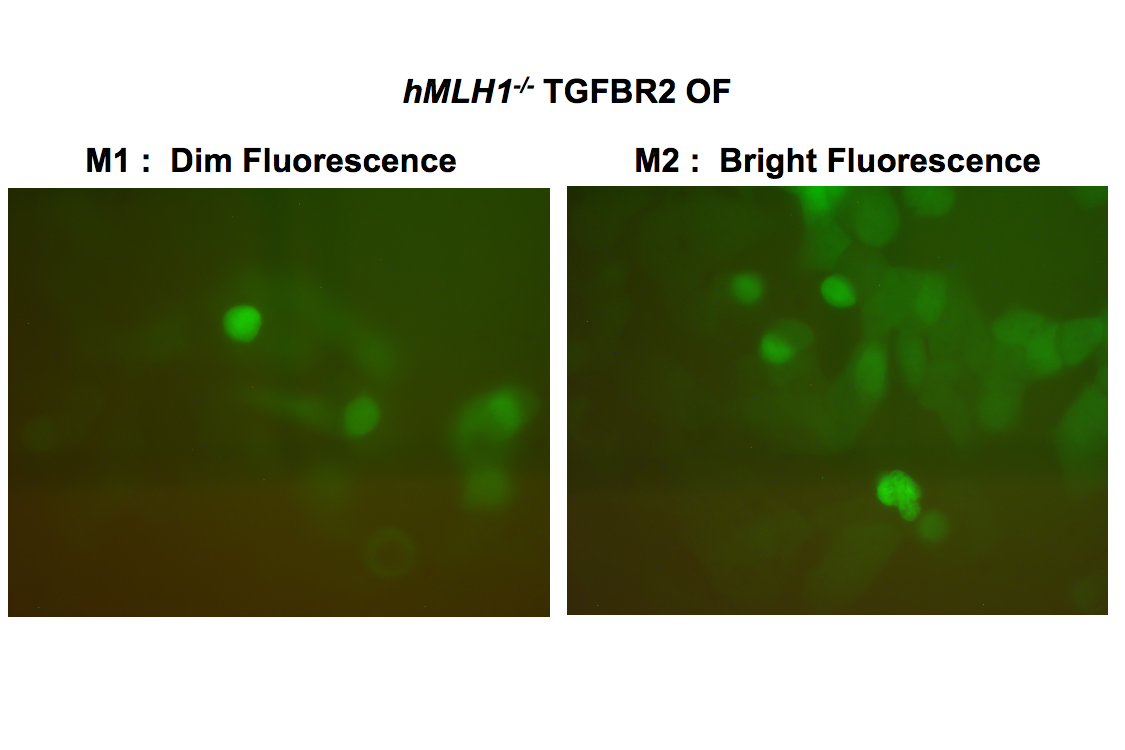
**

**Fig. S1. M2 cells from *hMLH1^-/-^* TGFBR2 OF showed brighter EGFP expression than counterpart M1 cells.** Fluorescent photos were taken as described in Fig. 2 legend.
